# A Pilot Study to Evaluate Genipin in *Staphylococcus aureus* and *Pseudomonas aeruginosa* Keratitis Models: Modulation of Pro-Inflammatory Cytokines and Matrix Metalloproteinases

**DOI:** 10.3390/ijms24086904

**Published:** 2023-04-07

**Authors:** Marcela Huertas-Bello, Jerson Andrés Cuéllar-Sáenz, Cristian Nicolas Rodriguez, Jesús Alfredo Cortés-Vecino, Myriam Lucia Navarrete, Marcel Yecid Avila, Elena Koudouna

**Affiliations:** 1 Department of Ophthalmology, Faculty of Medicine, Bogota DC, Universidad Nacional de Colombia, Bogotá 111321, Colombia; yhuertas@unal.edu.co (M.H.-B.); myavilac@unal.edu.co (M.Y.A.); 2 Grupo de Investigación Parasitología Veterinaria, Department of Animal Health, Faculty of Veterinary Medicine and Zootechnics, Bogota DC, Universidad Nacional de Colombia, Bogotá 111321, Colombia; jeacuellarsa@unal.edu.co (J.A.C.-S.); jacortesv@unal.edu.co (J.A.C.-V.); 3 Department of Microbiology, Faculty of Medicine, Bogota DC, Universidad Nacional de Colombia, Bogotá 111321, Colombia; cnrodriguezp@unal.edu.co (C.N.R.); mlnavarretej@unal.edu.co (M.L.N.); 4 Structural Biophysics Group, School of Optometry and Vision Sciences, Cardiff University, Cardiff CF24 4HQ, UK

**Keywords:** genipin, infectious keratitis, matrix metalloproteinases, pro-inflammatory cytokines, corneal collagen crosslinking, corneal crosslinking in infectious keratitis, bacterial keratitis, anti-inflammatory

## Abstract

Infectious keratitis is a vision-threatening microbial infection. The increasing antimicrobial resistance and the fact that severe cases often evolve into corneal perforation necessitate the development of alternative therapeutics for effective medical management. Genipin, a natural crosslinker, was recently shown to exert antimicrobial effects in an ex vivo model of microbial keratitis, highlighting its potential to serve as a novel treatment for infectious keratitis. This study aimed to evaluate the antimicrobial and anti-inflammatory effects of genipin in an in vivo model of *Staphylococcus aureus* (*S. aureus*) and *Pseudomonas aeruginosa* (*P. aeruginosa*) keratitis. Clinical scores, confocal microscopy, plate count, and histology were carried out to evaluate the severity of keratitis. To assess the effect of genipin on inflammation, the gene expression of pro- and anti-inflammatory factors, including matrix metalloproteinases (MMPs), were evaluated. Genipin treatment alleviated the severity of bacterial keratitis by reducing bacterial load and repressing neutrophil infiltration. The expression of interleukin 1B (IL1B), interleukin 6 (IL6), interleukin 8 (IL8), interleukin 15 (IL15), tumor necrosis factor-α (TNF-α), and interferon γ (IFNγ), as well as MMP2 and MMP9, were significantly reduced in genipin-treated corneas. Genipin promoted corneal proteolysis and host resistance to *S. aureus* and *P. aeruginosa* infection by suppressing inflammatory cell infiltration, regulating inflammatory mediators, and downregulating the expression of MMP2 and MMP9.

## 1. Introduction

Infectious keratitis is an infection of the cornea, the optically clear tissue that covers the front of the eye. It is the fourth leading cause of blindness globally and is responsible for ~5% of all cases and 10% of avoidable visual impairment in developing countries [1,2,3]. According to the World Health Organization, approximately 4.2 million people have cornea-related blindness or visual impairment [4]. With an annual estimate of 1.5–2.0 million cases of monocular blindness caused by corneal opacity, which is primarily caused by infectious keratitis, it is considered an ophthalmologic emergency. It has recently been recognized as a “neglected tropical disease” [5,6]. Significantly, vision impairment and blindness are associated with decreased educational and employment prospects, increased risk of death, and possess an enormous global health, social and economic burden causing annual costs of at least US $244 billion [4,5,7].

The major risk factors of infectious keratitis include contact lens wear, ocular trauma, post-corneal surgery (such as keratoplasty), and systemic diseases (diabetes, immunosuppression), among others [8,9,10,11]. It can be caused by various microorganisms, including bacteria, fungi, parasites, and viruses [3,5,12]. Bacterial keratitis is the most common type of corneal infection [8,9,13,14,15]. The most reported causative bacteria include *Staphylococcus aureus* (5–36%; *S. aureus*) and *Pseudomonas aeruginosa* (5–24%; *P. aeruginosa*) [5,16].

Severe infectious keratitis is caused by various virulence factors, such as bacterial toxins and enzymes produced during infection, and an overwhelming host immune inflammatory response that can contribute to severe tissue damage [16,17]. For instance, ongoing recruitment of neutrophils and other inflammatory cells results in excessive secretion of oxidants, proteolytic enzymes such as matrix metalloproteinases (MMPs), and inflammatory mediators that attack and damage the host structures [18,19]. Several studies have detected a cytokine storm-like phenotype in patients with corneal inflammation that compromise corneal transparency and induce swelling and neovascularization [20]. For example, interleukins (ILs) IL1B, IL6, and IL8 were reported to be significantly elevated in patients with bacterial keratitis [21,22,23]. Tumor necrosis factor-α (TNF-α) has been associated with the immunopathogenesis of uveitis, herpetic stromal keratitis, and corneal response to injury [24,25,26,27]. Therefore, a balanced host response to infection is vital in managing infectious keratitis, and effectively regulating the cytokine storm is critical to prevent tissue perforation and preserving vision. 

Broad-spectrum topical antimicrobial treatment is the gold standard [2]. However, visual outcomes are often poor, secondary to corneal melting, scarring, and perforation [14,28,29]. In cases of severe unresponsive keratitis, corneal transplantation is often the last therapeutic alternative; yet this is associated with increased risks of graft rejection/failure [30,31]. Organ shortage, with only one in 70 patients worldwide having access to it, is an additional universal problem [32]. Medical management and treatment of corneal infections are challenging, often with little success, the etiology of which is multifactorial. The increasing trend of antimicrobial resistance further compounds clinical management, and recent studies have highlighted antimicrobial resistance in ocular infections [28,33,34,35,36,37]. Consequently, there is an urgent need to develop novel, unconventional therapeutic approaches to treat and manage infectious keratitis. 

Genipin, a natural crosslinking agent, has received significant attention because of its biocompatibility, stability, and safety [38,39,40,41,42,43]. “Genipin is a naturally occurring iridoid compound extracted from the Gardenia jasminoides Ellis plant” [44]. Genipin has been reported to have various pharmacological actions, such as antimicrobial [45,46], anticancer [47,48,49], anti-inflammatory [50,51], hepatoprotective [48], and neurotrophic effects [52]. Its great potential as a natural crosslinking agent for biomaterials was first noted when genipin was compared to glutaraldehyde and demonstrated comparable crosslinking ability but significantly lower toxicity [53,54,55]. While it is well-known that genipin can link free amino groups of lysine or hydroxylysine residues of different polypeptide chains via monomeric or oligomeric crosslinks in collagen, this mechanism is complex and not fully understood yet [40,42,54,56,57]. 

Previously, we demonstrated the effects of corneal genipin crosslinking in ex vivo and in vivo models with minimal toxicity [45,58,59]. Several studies on sclera have also shown successful stiffening and biocompatibility in different animal models [55,60,61,62,63,64], supporting the prospect of sclera genipin crosslinking for the treatment of glaucoma and myopia. In addition, genipin has anti-inflammatory [44,50,63,65], antioxidant [52,66], and antibacterial/ antifungal properties [45,46]. Genipin has also been shown to delay corneal stromal melting and increase tissue resistance to enzymatic digestion [67]. 

In the present study, we sought to evaluate genipin for treating and managing bacterial keratitis using an in vivo model of *S. aureus* and *P. aeruginosa* corneal keratitis, with a particular focus on its effect in regulating the host immune response to infection. To address this, the clinical signs of infection, tissue morphology, bacterial cell viability, growth, infiltration of immune cells, and secretion of several inflammatory mediators at the site of infection were examined. Our study demonstrated that genipin has the potential to increase tissue resistance and halt corneal melting and perforation, and, importantly, modulate the host inflammatory response in response to bacterial infection. This highlights the unexploited potential of genipin to stimulate the resolution of infection-associated inflammatory response in infectious keratitis, which in combination with its antimicrobial and crosslinking properties, makes it a strong candidate as a potential novel therapeutic algorithm for the treatment and management of infectious keratitis and other ocular inflammatory diseases. 

## 2. Results

### 2.1. Genipin Alleviates the Severity of Bacterial Keratitis—Improved Management of Descemetocele and Corneal Perforation after Topical Genipin Treatment

Approximately six hours after bacterial inoculation with either *S. aureus* or *P. aeruginosa* and before the rabbits received any treatment, all infected corneas demonstrated signs of bacterial keratitis, and the mean slit-lamp examination (SLE) score was not statistically different between the groups. The average clinical scores at different time points of the induced keratitis for each treatment group are presented in Table 1. 

**Figure 1:**
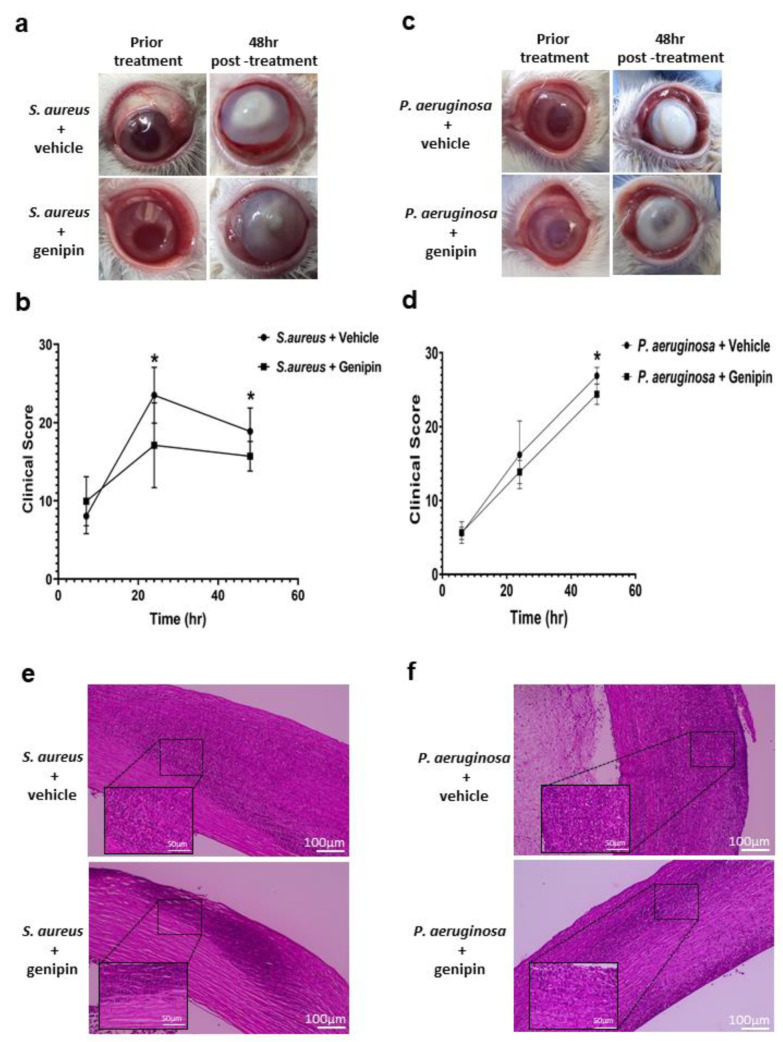
Genipin treatment ameliorates clinical manifestations of bacterial keratitis and regulates inflammatory cell infiltration. (**a**,**c**) Ocular surface observation and examination. Representative photographs of corneas infected with *Staphylococcus aureus (S. aureus)* or *Pseudomonas aeruginosa (P. aeruginosa)* prior to treatment and 48 h post-treatment with either vehicle solution or genipin. (**b**,**d**) Clinical scores at different times after bacterial inoculation. The data are the mean ± standard deviation of the mean, *n* = 5, * Statistically significant, *p* ≤ 0.05. (**e**,**f**) Histological analysis of infected corneas. Hematoxylin and eosin staining were used to examine the pathological changes at 48 h post-treatment. Representative images of five corneas per group.

In the *S. aureus* keratitis study, severe keratitis was developed in the vehicle-treated group, characterized by chemosis, corneal infiltrate, and stromal melting with hypopyon (Figure 1a). In addition, corneal perforation occurred in one of the five cases of vehicle-treated eyes. The pathological changes and disease severity of the genipin-treated group were milder with less infiltrate, chemosis, and ocular surface inflammatory changes (Figure 1a). There was a significant difference in signs of infection and the average clinical scores between the vehicle-treated eyes versus the genipin-treated eyes after 24 h (*p* = 0.0852, Table 1, Figure 1b) and 48 h post-inoculation (*p* = 0.0629, Table 1, Figure 1b).

Regarding the *P. aeruginosa* keratitis study, eyes in the vehicle-treated group displayed a more vigorous reaction with exacerbated corneal infiltration and intense ocular inflammation. Specifically, three out of five cases showed corneal perforation, and the appearance of descemetocele was noted in four of five cases (Figure 1c). In the genipin-treated group, although severe corneal infiltration and inflammatory response in the anterior chamber were also observed, only one eye out of five presented a descemetocele, and none of the corneas in this group were perforated. Genipin treatment resulted in a significantly reduced clinical score compared to the vehicle-treated control at 24 h *n* = 5, *p* = 0.0215, Table 1, Figure 1d) and 48 h post-inoculation (*n* = 5, *p* = 0.0014, Table 1, Figure 1d).

### 2.2. Decreased Inflammatory Cell Infiltration in Genipin-Treated Eyes 

Histopathological analysis of the vehicle-treated corneal tissue sections for both the *S. aureus* and *P. aeruginosa* keratitis showed that the corneal stroma was mainly homogenized with necrotic lamellae and significant cell infiltration at the site of infection (Figure 1e,f). Genipin-treated corneas observably showed reduced cell infiltrate and alleviated corneal edema (Figure 1e,f).

**Figure 2:**
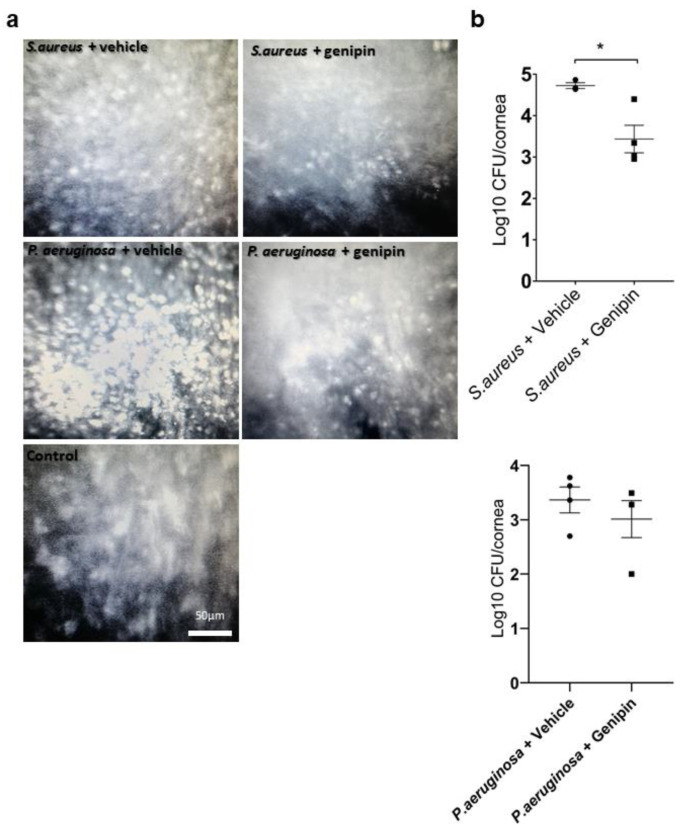
Genipin decreases bacterial growth in the *S. aureus* keratitis model. (**a**) Confocal microscopy images of the corneal stroma of infected corneas with *S. aureus* and *P. aeruginosa* treated with either vehicle solution or genipin at 48 h post-treatment. Genipin treatment demonstrated visible difference in the number of bacterial and inflammatory cells infiltration in the corneal stroma. No infiltrates were observed in the control corneas. Scale bar = 50 µm (**b**) The number of viable bacterial colonies in the infected corneas, examined by colony-forming unit (CFU) analysis, was expressed as log and plotted for each treatment group (*n* = 4 for each experimental group). Error bars represent standard deviation. * Statistically significant (*p* < 0.05).

Moreover, confocal microscope images of the *S. aureus* and *P. aeruginosa* infected vehicle-treated corneas demonstrated the deposition of hyper-reflective, round-shaped structures within the stroma, presumed to represent a mixture of bacteria and immune cells such as lymphocytes and plasma cells (Figure 2a). These structures appear irregular in shape and to a lesser density in the stroma of the genipin-treated corneas. Moreover, fine string-like structures interspersed between keratocytes appear, which likely correspond to crosslinked collagen fibers. These data suggest that genipin treatment reduces the inflammatory cell infiltration at the infected area in a rabbit *S. aureus* and *P. aeruginosa* rabbit model. 

### 2.3. Microbial Log Reduction

To evaluate the antimicrobial effect of genipin, the viable corneal bacterial load was investigated. For experimental *S. aureus* keratitis, eyes treated with genipin produced a significant decrease in the number of colony forming units (CFUs) per cornea compared to vehicle-treated eyes by almost two orders of magnitude, representing a 99% reduction in the *S. aureus* population. Specifically, treatment with genipin resulted in an average log 3.42 ± 0.65 CFU/cornea compared to the vehicle-treated group, which averaged a log 5.64 ± 0.93 CFU/cornea (*p* = 0.0142; Figure 2b). In the *P. aeruginosa* keratitis, treatment with genipin did not produce a significant reduction in the number of CFUs per cornea (Average log 3.01 ± 0.68 CFU/cornea) relative to the vehicle-treated group (Average log 3.42 ± 0.55 CFU/cornea; *p* = 0.570, Figure 2b). 

### 2.4. Genipin Regulates the Host’s Immune Response to Infection

**Figure 3:**
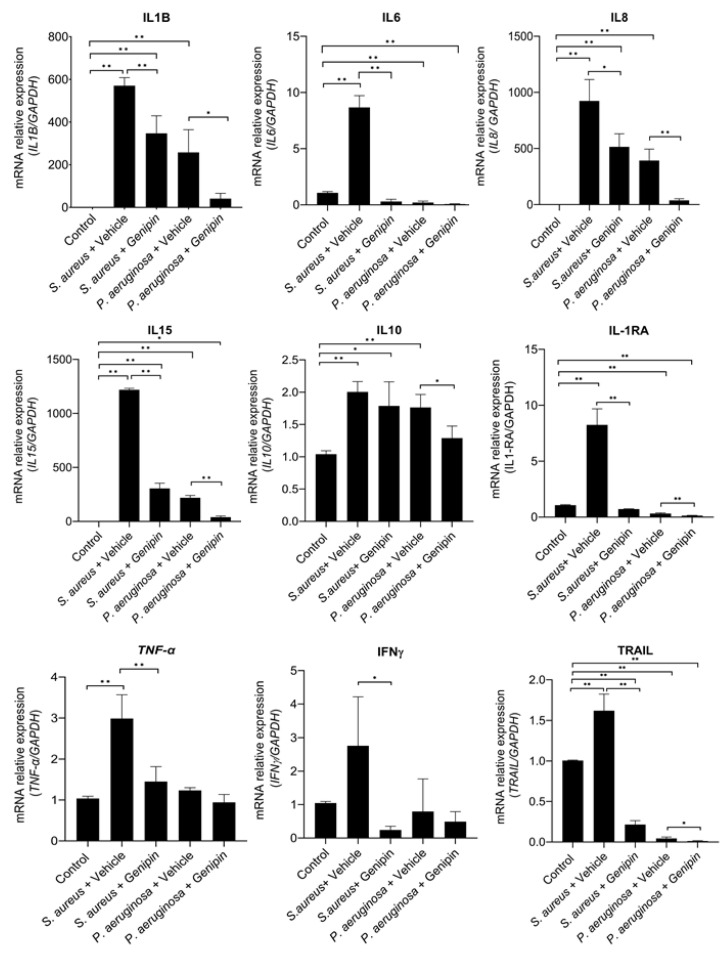
Genipin decreases the expression of inflammatory cytokines in the *S. aureus* and *P. aeruginosa* rabbit corneal keratitis model. Infected corneas were treated with either vehicle solution or genipin for 48 h. Total RNA was extracted and the mRNA expression levels of interleukin 1B(IL1B), interleukin 6 (IL6), interleukin 8 (IL8), interleukin 10 (IL10), interleukin 15 (IL15), interleukin 1 receptor antagonist (IL-1Ra), tumor necrosis factor-α (TNF-α), interferon γ (IFNγ) and tumor necrosis factor-related apoptosis-inducing ligand (TRAIL) were detected with qPCR. Data are average mRNA expression levels relative to GAPDH ± standard deviation. A total of five corneas were used for each experimental group and all samples were examined in triplicates. * *p* < 0.05, ** *p* < 0.01, comparing bracketed groups.

To evaluate the role of genipin in the inflammatory response in rabbits with *S. aureus* and *P. aeruginosa* keratitis, the expression of pro-inflammatory mediators and anti-inflammatory factors were assessed. The expression levels of cytokines, chemokines, chemokine receptors, and MMPs were highest following induction of infectious keratitis with either *S. aureus* or *P. aeruginosa*, in comparison to the healthy control, indicating that bacterial keratitis triggered a host inflammatory response (Figure 3). Of particular interest, these inflammatory factors’ gene expression levels demonstrated remarkable differences between genipin-treated and vehicle-treated groups for both keratitis models, *S. aureus* and *P. aeruginosa* keratitis (Figure 3). Specifically, the levels of IL1B, 1L8, and IL15 were found to be significantly decreased in genipin-treated corneas in the *S. aureus* (*p* = 0.005 for IL1B, *p* = 0.019 for IL8, *p* = 0.001 for IL15) and the *P. aeruginosa* (*p* = 0.013 for IL1B, *p* = 0.001 for IL8, *p* = 0.001 for IL15) keratitis model. In the *S. aureus* keratitis study, treatment with genipin revealed a significant downregulation of IL6, TNFα, and interferon γ (IFNɣ) compared to the vehicle-treated group (*p* = 0.001 for IL6, *p* = 0.008 for TNFα, *p*= 0.025 for IFNɣ). The expression levels of these cytokines were also decreased in the *P. aeruginosa* keratitis model in the genipin-treated group as opposed to vehicle-treated corneas; however, they showed no statistically significant difference. *S. aureus* and *P. aeruginosa* infection increased the expression of IL10, and this elevation of IL10 expression was significantly dampened with genipin treatment in the *P. aeruginosa* study (*p* = 0.025). The expression of IL-1RA was also significantly attenuated by genipin treatment in the *S. aureus* (*p* = 0.001) and *P. aeruginosa* (*p* = 0.002) study. 

**Figure 4:**
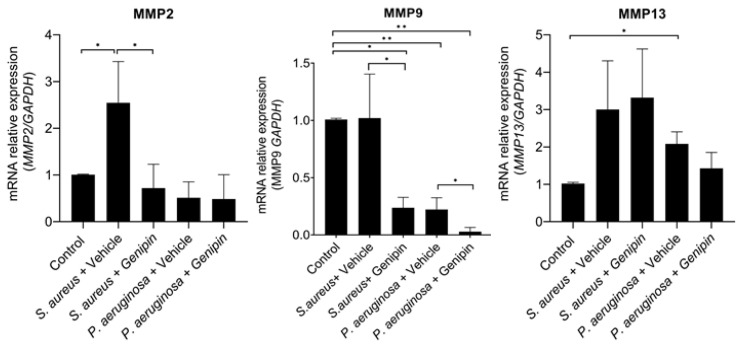
The expression of MMPs in the infected corneas was decreased in infected corneas treated with genipin. Corneas infected with *S. aureus* or *P. aeruginosa* were treated with either vehicle solution or genipin for 48 h. Total RNA was extracted and the mRNA expression levels of MMP2, MMP9, and MMP13 were evaluated with qPCR. Data are average mRNA expression levels relative to GAPDH ± standard deviation. A total of five corneas were used for each experimental group and all samples were examined in triplicates * *p* < 0.05, ** *p* < 0.01, comparing bracketed groups.

MMPs have an essential role in tissue remodeling and wound healing. In comparison to the vehicle-treated group, genipin treatment significantly decreased the expression levels of MMP9 in both *S. aureus* (*p* = 0.013) and *P. aeruginosa* keratitis (*p* = 0.022) (Figure 4). Similarly, the expression values of MMP2 were reduced following treatment with genipin, but this was only found to be statistically significant in the *S. aureus* model (*p* = 0.021) (Figure 4). No statistically significant difference was noted in the expression levels of MMP13 between genipin- and vehicle-treated groups (Figure 4). 

The tumor necrosis factor-related apoptosis-inducing ligand (TRAIL) expression levels were also evaluated (Figure 3). In the *S. aureus* study, the gene expression levels of TRAIL significantly increased upon infection, which was diminished by genipin treatment (*p* = 0.01). *P. aeruginosa* infection also decreased the expression of TRAIL in comparison to the healthy cornea, and this was significantly attenuated with genipin treatment (*p* = 0.018).

## 3. Discussion

Infectious keratitis is an important cause of blindness due to the difficulty of treatment and progression to corneal ulcers caused by excessive degradation of collagen in the corneal stroma and perforation requiring cornea transplant surgery as a last resort [68,69]. An essential aspect of the pathogenesis of bacterial keratitis is the host’s inflammatory response, as uncontrollable and persistent infiltration of inflammatory cells may lead to delayed corneal wound healing, corneal opacity, and even result in corneal perforation, and vision loss. Although corneal transplantation is highly successful in low-risk cases, in the context of an inflamed eye, it has little success rate [5,70,71]. The dramatic increase in multidrug-resistant microbes is an additional challenge in managing the disease using antimicrobials, further emphasizing the urgent need for new therapeutic approaches for keratitis [72]. Therefore, it is necessary to establish new therapeutic strategies that display antimicrobial and anti-inflammatory properties and protect tissue from enzymatic digestion. Our previous studies showed that genipin, a natural crosslinking agent, has antibacterial properties and reduces the colonization and proliferation of *S. aureus* and *P. aeruginosa* in an ex vivo corneal infection model [46]. We also recently demonstrated that genipin is associated with minimal corneal toxicity, effective crosslinking activities, and, importantly, that it increases corneal stromal resistance to enzymatic digestion [45,67].

Corneal local response to infection and bacterial toxins includes an inflammatory process with infiltration of neutrophils, granulocytes, monocytes, and macrophages at the site of infection for pathogen clearance [68,73,74,75]. This inflammatory response is associated with elevated expression of pro-inflammatory cytokines (TNF-a, IL1b, IL1a, IL6) and the generation of reactive oxygen species that can worsen the injury, alter the normal function of endogenous macrophages and stem cells by avoiding tissue regeneration and ultimately affecting corneal transparency and normal tissue structure-function relationship [76,77,78,79]. Retarding corneal melting in the setting of infectious keratitis could potentially minimize the risk of corneal ulceration, melting, and perforation. Thus, this study sought to evaluate the effectiveness of genipin for the management and treatment of infectious keratitis in an *S. aureus* and *P. aeruginosa* rabbit keratitis model. The ability of genipin to modulate the host’s immune response to infection was also investigated. To our knowledge, there is no previous study examining the use of genipin in vivo on an *S. aureus* and *P. aeruginosa* rabbit model of infectious keratitis. Our data revealed that although a two-dose regimen treatment of genipin has a limited effect on bacterial cell viability and growth and a higher dose is required, it plays an essential role in the regulation of the host’s immune response to infection, a requisite to restrict bystander damage limiting tissue disruption resulting from an inflammatory response. Most importantly, these data suggest genipin as a potential means for haltering corneal ulcers, melting, and perforation. Its plausible application as an adjuvant therapy for the treatment and management of infectious keratitis, therefore, warrants further future investigation.

To explore the therapeutic potential, genipin was topically applied to infected rabbit corneas. Genipin treatment *versus* vehicle-treated control reduced ulcer area, and consistently, clinical scores of genipin-treated corneas were lower than that in vehicle-treated, indicating that genipin may play a protective role in the progression of bacterial keratitis. One emphasis of future research is to elucidate the corneal depth penetration properties of genipin in the healthy and infected cornea and importantly, to better optimize dosage schedule delivery and application. Moreover, colony-forming unit analysis demonstrated that the administration of genipin reduced the number of live bacteria in infected corneas in comparison to the control group. These results support previous studies that showed the antimicrobial effect of genipin [45,46,65]. Interestingly, our findings demonstrated that genipin exerts a more significant effect against *S. aureus* than *P. aeruginosa* with respect to eliminating bacterial growth in the cornea. In the *S. aureus* keratitis study, a two-times topical administration of genipin significantly reduced the number of viable bacteria in the infected corneas. In the *P. aeruginosa* study, although genipin treatment resulted in a reduction in the viable colonies per cornea, this was not significant. These results disagree with previous in vitro studies that suggested genipin to be more potent against *P. aeruginosa* than *S. aureus.* One explanation for this is that in an in vivo setting, *P. aeruginosa* is a more virulent ocular pathogen that can rapidly progress and cause corneal perforation in just 72 h [80]. Another important point to note is that, in the previous in vitro studies, bacterial inoculation was carried out in a different experimental approach, namely bacteria inoculated in scalpel-wounded corneas versus instrastromally injected bacteria in an in vivo environment [45]. Despite this, the marked reduction in bacterial killing after genipin treatment is notable in both models of infectious keratitis and highlights the need for optimizing the dosage, frequency, and duration of the therapy for each microorganism. In addition, further investigations are necessary to fully elucidate the antimicrobial efficacy of genipin, especially in combination with other antimicrobials. The exact mechanism of action of genipin remains elusive and future studies are indeed required to more fully understand how genipin exerts its anti-microbial effects. However, taking into consideration its natural crosslinking properties [40,42], a plausible microbial killing mechanism is the crosslinking of various bacterial proteins present at the surface of the pathogen, such as, for example, peptidoglycan, lipoteichoic acid, and lipopolysaccharide [81]. As a result, this would interfere with normal cellular processes such as cell division, proliferation, and growth. Further, interaction of genipin with surface membrane proteins would also likely interfere with key metabolic functions, rendering bacterial survival and replication impossible. 

The host’s inflammatory response plays a vital role in the progression and management of corneal infectious keratitis. From one point of view, the inflammatory response is indispensable, in which polymorphonuclear leukocytes, macrophages, and inflammatory cells are recruited in the infected area and secrete numerous cytokines and chemokines to attenuate the infection and defend the microorganism [82] to enhance the inflammatory process and immune cell infiltration. From another point of view, excessive and markedly persistent inflammation may delay or prevent wound healing and give rise to tissue perforation and potentiate corneal opacity and vision loss [83]. Tight regulation and resolution of the host’s inflammatory response, thus, plays an indispensable role in the management and treatment of infectious keratitis [72,84]. In our study, in vivo confocal microscopy and histological analysis showed a diminished flux of polymorphonuclear cells at the site of infection, particularly in the *S. aureus* keratitis model, suggesting that genipin reduces inflammatory cells infiltration and ameliorates corneal damage. Consistently, previous studies also reported that genipin regulates inflammasome activation and reduces inflammatory cell infiltration and fibroblast proliferation in wound healing of diabetic rats [85]. Interestingly, previous studies evaluating the immune events in response to non-crosslinked and crosslinked liver matrices in a rat model of abdominal wall muscle repair showed that crosslinking with genipin reduced host immune reactions with moderate of lymphocyte and neutrophil infiltration [63].

As stated above, an important aspect of the pathogenesis of infectious keratitis is the secretion of inflammatory cytokines [16,20,67,75,86,87,88]. Our results demonstrated that genipin treatment regulates the host cellular and humoral immune response and, thus, impacts the secretion of inflammatory mediators and factors at the side of infection. Genipin treatment significantly diminished the expression of IL1β, IL8, IL15, and IL-1RA in both *S. aureus* and *P. aeruginosa* keratitis models. Furthermore, genipin treatment significantly repressed the production of TNF-α and IFNɣ in *S. aureus* infected corneas. These results align with previous studies illustrating the anti-inflammatory properties of genipin [48,50,51,65,85,89]. Wang and colleagues showed that genipin exerts protective effects on LPS-induced inflammation by repressing LPS-induced TNF-α and IL1β expression in BV2 microglial cells in brain tissues [50]. This study forms a baseline for future detailed investigations into the impact of genipin treatment on the regulation of the host immune response in corneal keratitis. One limitation of this study is the use of solely one reference gene in the gene analysis experiments [90]. We recognize the requirement for a quantitative approach to better understand the control of gene expression of these inflammatory factors and their precise protein levels in the infected cornea. Larger sample size studies would be needed in the future to assess the gene and protein expression levels of inflammatory mediators and MMPs at the infected cornea and identify more precisely the underlying mechanism by which genipin exerts its regulatory influences. Larger sample size studies would be needed in the future to assess the gene and protein expression levels of inflammatory mediators and MMPs at the infected cornea and identify more precisely the underlying mechanism by which genipin exerts its regulatory influences. 

Another critical aspect of infectious keratitis is corneal ulceration, defined as the destruction of the normal tissue architecture associated with the degradation and melting of stromal collagen. Besides bacterial collagen degrading enzymes that directly mediate collagen degradation, corneal epithelial and stromal cells are also responsible for the secretion of MMPs, which largely participate in tissue degradation and remodeling. Increased proteolytic activity has been reported to be upregulated in patients with corneal melting, infectious keratitis, and corneal burning and are linked to epithelial barrier dysfunction, as well as an excessive inflammatory response by the activation of IL6 [63]. Augmented secretion and activity of MMPs, particularly MMP2 and MMP9, have been proposed to contribute to excessive corneal ulceration and worsening clinical outcomes during *P. aeruginosa* infection [18,91]. In this study, genipin treatment significantly reduced the gene expression of MMP2 and MMP9 and could potentially attenuate excessive tissue degradation by MMPs. In this context, our previous studies demonstrated that genipin enhances tissue stabilization and protection from bacterial enzymatic digestion [67,92]. Taken together, these findings suggest that genipin treatment could ameliorate the destruction of corneal collagen and structure and contribute to better management of the disease progression and hence, better clinical outcome. 

In conclusion, in this study the effects of genipin in *S. aureus* and *P. aeruginosa* infectious keratitis models were evaluated in vivo. Remarkably, genipin treatment was shown to modulate the host inflammatory response to infection and could potentially halt tissue degradation, important prerequisites for tissue repair and management. Genipin through its crosslinking, antimicrobial, and anti-inflammatory action could inhibit the microbial invasion of the cornea, reduce inflammatory cell recruitment in infected sites, and decrease the expression of inflammatory factors, and it is therefore a promising compound, providing a novel direction for the management and treatment of infectious keratitis. Data presented here are supportive of the role for genipin in the management of bacterial keratitis, with future studies further elucidating its effect as an adjuvant therapy, in combination with other antibiotics, or as a stand-alone therapy, required to fully unravel its therapeutic potential. Further investigations should be aimed at elucidating the effects of genipin in combination with other antibiotics and other anti-inflammatory compounds. Clearly understanding the anti-microbial and anti-inflammatory properties of genipin and the underlying mechanisms has both significant clinical and scientific implications as it can lead to the establishment of novel therapeutic approaches for the management and treatment of corneal keratitis and other ocular inflammatory disorders.

## 4. Materials and Methods

### 4.1. Study Design

This study was performed in accordance with the institutional guidelines and tenets of the Code of Practice for the Housing and Care of Animals Bred, Supplied, or Used for Scientific Purpose Act 1986. Ethical approvals for this study were granted by the Faculty of Medicine ethical committee (approval act: 019-212) and by the Faculty of Veterinary Medicine and Zootechnics ethical committee (approval act: 01- 2021, CB-FMVZ-UN-004-2021). All the experiments were carried out under veterinary supervision. 

New Zealand white rabbits weighing between 2.5–3.5 kg were used in this study. The rabbits were housed in the Animal Facility of the Faculty of Veterinary Medicine of the National University of Colombia in a private room. We calculated that the sample size in each treatment group should be 3 at an alpha of 0.05 and a power of 0.80. The animals were supervised by qualified personnel, and insulation, temperature, ventilation, noise control, and natural light in the holding room were granted. After the acclimatization period (15 days) and before the initiation of the study, the animals were inspected for any ocular damage or health condition to ensure that they were suitable for inclusion in the study. In separate experiments, in vivo studies were performed on rabbit eyes with infectious keratitis caused by either *S. aureus* or *P. aeruginosa.* The rabbits were equally divided into an *S. aureus* keratitis group (*n* = 10; male rabbits) and a *P. aeruginosa* keratitis group (*n* = 10; female rabbits). The staphylococcal keratitis group was subdivided into two groups, to be treated with either genipin (*n* = 5) or vehicle as a treatment control (*n* = 5). Similarly, the pseudomonal keratitis group was subdivided into two groups, one to be treated with genipin (*n* = 5) or vehicle (*n* = 5). Among the five animals that received vehicle as a treatment, the contralateral, uninfected, and untreated eyes served as a healthy control (*n* = 5 for *S. aureus* group and *n* = 5 for *P. aeruginosa* group). Half cornea was used for bacterial plating in order to estimate the number of viable microbes per cornea. The other half cornea was then divided into two quarters, one of which was used for histology and the other for RNA isolation and quantification of the gene expression of several inflammatory mediators.

### 4.2. Bacterial Strains

Bacterial strains of *S. aureus* (ATCC 25923) and *P. aeruginosa* (ATCC 27853), purchased from the American Type Culture Collection (Labcare de Colombia Ltd., Cota, Colombia), were used to constitute corneal infectious keratitis as described previously [46]. Bacteria were grown on brain-heart infusion (BHI) agar (Suministros Clinicos ISLA S.A.S, Bogotá, Colombia) overnight at 37 °C and maintained at 4 °C. One day before the in vivo studies and infection of the corneas, one colony was cultured into fresh BHI agar and incubated at 37 °C overnight. On the day of the experiment, a bacterial suspension of approximately 1.5 × 10^8^ colony-forming units per milliliter (CFU/mL) was prepared using a 0.5 MacFarland standard. 

### 4.3. Induction of Keratitis and Treatment Regimen

Before surgery, animals were anesthetized with intramuscular doses of ketamine^®^ (Ketamina 50%, Holliday-Scott S.A., Buenos Aires, Argentina; distributed by Gabrica S.A.S., Colombia), and xylazine (Rompun^®^Xilazina 2%, Bayer S.A., Colombia) (35 mg/kg and 5 mg/kg). One drop of proparacaine hydrochloride (Alcaine, Alcon, Ft, Fort Worth, TX, USA) was also topically administered in the eye for corneal anesthesia. The bacterial keratitis was induced as previously described [46]. Briefly, once animals were anesthetized, they were placed beneath a stereoscopic microscope, and the left cornea per rabbit was intrastromally injected with 0.1 mL bacterial suspension containing 1.5 × 10^7^ colony-forming units using a sterile BD Ultra-Fine Insulin Syringe—30 G 1 cc 1/2 (BD, Farmalisto, Bogotá, Colombia). The experimental keratitis for *S. aureus* and *P. aeruginosa* was allowed to proceed untreated for 6 h, and after that, topical therapy was initiated. *Eyes received topically two 100 µL doses of genipin, 3.40 mg mL^−1^ at* 6 h and 24 h after bacterial inoculation or vehicle. Tramadol (5 mg/kg; Genfar, S. A., Colombia) was administered subcutaneously to the rabbits to manage mild to moderate pain. The animals were sacrificed 48 h after bacterial inoculation and euthanized by intravenous injection of euthanex^®^ (1 cc/5 kg; Invet S.A., Bogotá, Colombia), and subsequently, the corneas were harvested using an aseptic technique.

### 4.4. Clinical Examination

All the eyes were clinically examined before bacterial inoculation and at 6, 24, and 48 h post-inoculation. Clinical appearance was evaluated and graded for severity of infection according to the clinical parameters based on the McDonald–Shadduck scoring system by two blinded ophthalmologists, as shown in Table 2 [93]. Corneal opacity degree, corneal opacity area, corneal ulceration, area of initial injury, redness of palpebral conjunctiva, redness of nictitating membrane, discharge, hypopyon, and chemosis or inflammation were graded on a scale ranging from 0 (none) to a maximum of 4 (severe). The sum of these parameters for each eye determined the SLE score of pathological changes. 

### 4.5. Confocal Corneal Evaluation

Infected, control, and treated corneas from the different groups (genipin-treated group versus vehicle-treated group) were evaluated at 48 h post-induction of bacterial keratitis. Confocal images were obtained with a Confoscan 4 (Nidek, Fort Lauderdale, FL, USA) using the 40× immersion objective. Cellular changes, infiltration, and corneal necrosis formation were evaluated. 

### 4.6. Histological Analysis

Corneal specimens were fixed in 10% formaldehyde, dehydrated with ethanol, and processed for paraffin embedding and histologic analysis. Tissue sections (5 μm thick) were stained by hematoxylin and eosin (H&E) and Gram stains and subsequently imaged using an Olympus microscope (Olympus, Tokyo, Japan) and a Canon EOS T4i Rebel camera (Canon Inc., Tokyo, Japan).

### 4.7. Bacterial Colony-Forming Unit Analysis—Plate Count

Corneal specimens were homogenized in 1 mL of sterile saline solution, and the homogenate was tenfold serially diluted. Then, 100 µL of each dilution was plated in BHI agar plates at 37 °C for 24 h. Colony enumeration was carried out, and CFU/cornea were expressed as base 10 logarithms, as described elsewhere [45]. 

### 4.8. Quantitative Real-Time Polymerase Chain Reaction

The inflammatory response of infected corneas was investigated by quantitative real-time polymerase chain reaction (RT-qPCR). A total of five corneal specimens were isolated per group, immediately immersed in RNA later solution (Thermofisher), and stored at −80 °C until RNA extraction. Total RNA was extracted using the Monarch total RNA extraction kit (New England, Biolabs, Boston, MA, USA). This was followed by quantification of the RNA concentration, transcription to cDNA, and amplification using the Luna One-step RT-qPCR universal kit (New England, Biolabs, Boston, MA, USA), as per manufacturer’s instructions. RT-qPCR was carried out (iCycler iQ; Bio-Rad, Hercules, CA, USA) with SYBR green PCR master mix (Bio-Rad). The specific primers listed in Table 3 were designed and validated in terms of efficiency. A computer program (version:2020.2 software; Bio-Rad) was used to visualize the data. The standard curve method was used to determine relative changes in gene expression levels, with GAPDH serving as the reference, which did not change significantly in our samples [94]. Experiments were performed in triplicates. 

### 4.9. Statistical Analysis

The clinical scores and the bacterial CFU data were compared between each treatment group using the two-sample t-test and the Mann-Whitney U test, respectively. The differences in relative gene expression data of all groups were determined using a one-way analysis of variance (ANOVA). Statistical significance was ascertained at *p* ≤ 0.05. 

## Figures and Tables

**Table 1:** Average slit-lamp examination (SLE) score at 6 h, 24 h, and 48 h of experimental groups (*n* = 5 per experimental group).

Microorganism	SLE Score Post-Inoculation ± SE		
	6 h	24 h	48 h
*S. aureus* + vehicle	8.05 ± 1.01	23.5 ± 1.60	20.7 ± 2.16
*S. aureus* + genipin	9.95 ± 1.40	17.1 ± 2.42 *	15.7 ± 0.85 *
*P. aeruginosa* + vehicle	5.69 ± 0.45	18.13 ± 0.92	27.25 ± 0.48
*P. aeruginosa* + genipin	6.0 ± 0.71	14.44 ± 0.50 *	24.88 ± 0.52 *

* Statistically significant, *p* < 0.05.

**Table 2:** Clinical parameters used in the slit-lamp examination score (McDonald–Shadduck scoring system).

A. Corneal Opacity Degree	Absence	0
	Visible Iris	1.5
	Iris details indistinguishable	3
	Anterior chamber invisible	4
B. Corneal Opacity Area	None	0
	≤ (not 0)	0.5
	>1/4 to <1/2	1
	>1/2 to <3/4	1.5
	>3/4	2
C. Corneal Ulceration	Normal curvature	0
	Protrusion or depression	1.5
	Perforation	3
D. Area of the initial injury	Same or lower	1.5
	Higher	3
E. Redness of nictitating membrane	Normal	0
	Slightly vascular dilation and edema	1
	Remarkable vascular dilation and redness of the entire membrane	2–4
F. Discharge	No discharge	0
	Any quantity	1
	Wet eyelids and eyelashes	2
G. Hypopyon	Not observed	0
	<1/4 of the corneal radius	1
	>1/4 to <1/2	2
	>1/2	3
I. Chemosis or inflammation	Not observed	0
	Clearly with eyelid disturbance	1
	Partially closed eyelids	2
	Closed eyelids	3

**Table 3:** Oligonucleotide Sequences for qRT-PCR.

Gene	Forward 5′-3′	Reverse 5′-3′
*IL-1RA*	GAAGTTGTGCCTGTCTTGTGTG	CCTCCTGGAAGTAGAACTTGGT
*IL-1b*	TGTTGTCTGGCACGTATGAGCTG	CTTCTTCTTTGGGTAACGGTTGGG
*IL-6*	CTGAAGAACATCCAACACCTGATC	CCTAACGCTCATCTTCCTAGTTTC
*IL-8*	ACACTCCACACCTTTCCATCC	CCTACGACAGATCCATGCAGT
*IL-10*	CCCGATCCTATTTATTTACCGAGC	GTTAGAAAGTGTGGTCAGGCACAG
*IL-15*	CTGTATCAGTGCAGGTCTTCC	CCTCCAGTTCCTCACATTCTTTGC
*TNF-a*	CTCCCAGGTTCTCTTCAGCGGTC	GTCCAGGTACTCAGGCTGGTTGA
*IFN-* *ϒ*	GCCAGGACACACTAACCAGAG	CCTCGAAACAGCGTCTGACT
*TRAIL*	CTGATCCTGATCTTCACAGTGCTCC	CTACTCTCTGAGGCCCTCTTTCTC
*MMP9*	TGGGCTTGGATCACTCCTCTG	CAGCTTGTTCCCTATCTCGGC
*MMP13*	CCAGATTTATCCTGATGGGCGTAC	CACTTGGGAATAGGCTTCCGC
*MMP-2*	TGGACCAGAGCACCATCGAG	GTGGAGCACCAGAGGAAGCC
*GAPDH*	GCGTGAACCACGAGAAGTATGACAAC	CAGTGGAGGCAGGGATGATGTTC

## Data Availability

All data are available upon request.
